# Stress as a Trigger for Relapses in IBD: A Case-Crossover Study

**DOI:** 10.4021/gr528e

**Published:** 2013-03-09

**Authors:** Susanna Jaghult, Fredrik Saboonchi, Jette Moller, Unn-Britt Johansson, Regina Wredling, Marjo Kapraali

**Affiliations:** a Karolinska Institutet, Department of Clinical Sciences, Danderyd Hospital, Sweden; b Red Cross University, Sweden; c Karolinska Institute, Department of Clinical Neuroscience, Division of Insurance Medicine, Sweden; d University of Stockholm, Stress Research Institute, Sweden; e Karolinska Institute, Department of Public Health Sciences, Division of Public Health Epidemiology, Sweden; f Sophiahemmet University College, Sweden

**Keywords:** Inflammatory bowel disease, Trigger factors, Stress, Case-crossover design

## Abstract

**Background:**

It is important to identify factors that influence the risk of relapses in inflammatory bowel disease. Few studies have been conducted and with limited methodology. This prospective case-crossover study, aims to examine whether perceived stress has a short-term acute effect, namely whether it acts as a trigger, on the risk of relapse in inflammatory bowel disease.

**Methods:**

Sixty patients with inflammatory bowel disease and in remission were included. The case-crossover design was employed, which is an epidemiological design developed to study triggers for acute events and diseases. To collect information regarding symptoms and potential trigger factors, such as perceived stress, a structured diary was constructed. The participants were instructed to fill in the diary daily during six months. Fifty patients completed the study.

**Results:**

The analysis showed an effect for high level of perceived stress. Being exposed to “quite a lot” of stress, yield an increase in risk for relapse during the forthcoming day (OR = 4.8, 95% CI 1.09 - 21.10). No statistically increased risk for lower levels of perceived stress was found, although elevated effect estimates were found for “some” stress.

**Conclusion:**

This study supports earlier findings regarding perceived stress as an important factor in triggering relapses in IBD. However, this is the first case-crossover study performed to explore the trigger risk of stress in this population. Further investigations with larger patient samples are needed to confirm the findings.

## Introduction

Inflammatory bowel diseases (IBD), including ulcerative colitis (UC) and Crohn’s disease (CD), are chronic diseases. The course of IBD is characterized by episodes of relapse and remission. Rectal bleeding, abdominal pain and diarrhoea are common symptoms of relapses [1]. Living with IBD and its unpredictable course can have a major impact on several aspects of life and require many lifestyle adjustments. Patients with increased disease activity experience more severe bowel symptoms, which in turn interfere with daily activities, but they also report more disease-related worries and poorer general well-being compared with patients in remission [2]. Studies have shown that patients with IBD rate their health-related quality of life (HRQOL) lower than the general population [3-6] and also that the level of disease activity is one of the most important explanations for decreased HRQOL[2, 4-8]. Consequently, it is very important to identify factors that influence the risk of relapses in IBD. Clinical care of these patients may be optimized if such factors can be controlled clinically or through patient’s self-care behaviour. However, few studies have been conducted so far, and methodology is limited. It has been suggested that a host of factors, such as diet, smoking, infection, antibiotics, and NSAID, influence the occurrence of relapses [9-12]. So far no studies have identified triggers for relapses in IBD, although during the last decade, psychological stress has repeatedly been reported as a potential trigger [9, 10, 13-16].

There is some evidence that perceived stress can trigger relapses, but the findings are inconclusive [9, 13-15]. Some studies measure life events as a proxy of perceived stress and do not report any association with changes in disease activity [17, 18]. Furthermore, experimental acute stress has been shown to induce systemic and mucosal proinflammatory responses, which could be a potential mechanism for relapses in UC [16]. Further, stress may have an effect on ion secretion, gut motility, inflammatory response and gut permeability [19-21]. Several combinations of stress response systems influence the inflammatory processes in IBD [22]. For example, stress mediates cytokine release and these cytokines may induce inflammatory activities [23].

When aiming to study the effect of stress on relapses in IBD, there are several methodological considerations to be dealt with. A large portion of the existing literature on stress and relapses has relied on mainly cross-sectional data, which inherently implies difficulties in drawing conclusions regarding causality. A relapse in IBD is a stressful event for the patient and in cross-sectional data it may be difficult to decide whether stress is the cause or a consequence of the relapse. Accordingly, there is a great need for prospective studies to explore what triggers relapses in IBD, and establish sequentiality with regard to stress and symptoms of relapses. The case-crossover design is an epidemiological tool which enables us to study triggers for acute events, and constitutes a valuable methodology for establishing causality between perceived stress and relapses in IBD [24]. To our knowledge, no case-crossover study on IBD has previously been reported.

The present study, a prospective case-crossover study, aims to examine whether perceived stress has a short-term acute effect, namely whether it acts as a trigger, on the risk of relapse in IBD.

## Methods

### Study design

We employed a case-crossover design which is an epidemiological design developed to study triggers for acute events and diseases [24]. The method departs from the assumption that if there are factors influencing the onset of an outcome, in this case a relapse in IBD, these would be present more often during a period just before the onset than during periods further from the onset [25]. Only cases were examined and were self-matched by serving as their own controls. Within each case, exposure frequency during the period prior to the onset of a relapse in IBD, the case period, was compared with exposure frequency during one or more control periods during which no relapses in IBD took place [24, 25].

### Study sample

The study sample consisted of patients identified, by a nurse specially trained in IBD, in a local register at the IBD clinic at Danderyd Hospital in Stockholm, Sweden. The local register contains information about all patients at the clinic with regard to diagnosis, localization of the disease, sex, age, and year of diagnosis. The information has been gathered by the nurses at the clinic for the purpose of creating a national quality registry. Those who were eligible for participation were patients with a diagnosis of UC or CD, who had suffered from the disease for less than two years, had no other chronic disease, had not undergone surgery due to IBD, and were in clinical remission. Clinical remission was defined based on the following criteria: no bowel symptoms associated with active disease, namely no diarrhoea or blood in stools, and no acute pharmaceutical treatment. Furthermore, the UC patients were to have an Ulcerative Colitis Disease Activity Index score of 2 or less [26], and CD patients were to have a Harvey-Bradshaw Index score of less than 5 [27].

After identification the patients were sent an invitation letter including information about the study. Patients who gave informed consent were given detailed information by telephone. Participation was voluntary, and the patients could withdraw from the study at any time. A total of 113 non-consecutive patients were invited to participate in the study, and 60 (53%) accepted and were enrolled. Of these 60 patients, 50 (84%) participated throughout the whole study follow-up.

### Data collection

To collect information regarding symptoms of IBD and a number of potential triggers, a diary was constructed (supplementary data). The potential triggers included were based on previous literature on possible triggers for relapse [9, 10]. Patients were instructed to fill in the diary every day during a follow-up period of 26 weeks and were asked to send in the completed diary pages every fourth week. If the pages were not sent in, the IBD nurse contacted the patient through telephone reminded about filling and sending in the missing pages and also inquired about the reason for not sending in. When including the patients in the study, they were informed to leave pages blank if they forgotten to fill in one day. Ten patients discontinued and stated reasons such as low motivation or forgetting to fill in the diary, or they experienced that it was not meaningful because they had no variation in the reported variables.

Before the main study was conducted, a pre-test study was carried out by getting two patients to test the designed diary. In addition, we wanted to test how acceptable it was to fill in the diary every day. Two patients filled in the diary during one month. After the pre-test study the diary was only slightly adjusted; for example, an open question was added to give the participants the opportunity to give other subjective important information.

### Outcome

Questions in the diary were used to identify relapses and concerned number of loose stools during daytime and night-time, occurrence of urgency, blood in stools, and abdominal pain. One question concerned the patients’ general well-being. The patients were also asked to state changes in medical treatment due to IBD or other illness.

Relapse of IBD was defined according to Truelove and Witt criteria, i.e. blood in stools, for patients with UC and for patients with CD in the colon [28]. For patients with CD in the small bowel, relapse was defined when reporting more than two consecutive days of abdominal pain. Medical files were scrutinized in order to check identified relapses.

Onset of a relapse was considered as the first day with a relapse. A relapse was classified as over when 14 consecutive days had passed with no blood in stools, no diarrhoea, or no abdominal pain.

### Exposure

Stress was assessed by single-item measure [29, 30] on a daily basis in the diary by answering the question: “Have you felt stressed today?”. Stress was measured on a five-graded response scale ranging from “not at all” (0), “a little” (1) “some” (2), “quite a lot” (3) to “a lot” (4).

### Statistical analysis

We selected two types of control information employing the matched-pair interval approach, which uses exposure status during a matched time period, for example, the same weekday as the day before the day of the onset of a relapse, but one week earlier. We also employed the usual frequency approach based on the frequency of exposed days during the control period.

The 1 - 7 days prior to the onset of a relapse were considered as the case period and the 8 - 14 days prior to the onset of a relapse were considered as the control period. Hazard periods of varying length were tested.

For estimation of odds ratios (OR) and 95% confidence intervals, conditional logistic regression was used in the matched-pair interval approach and standard Mantel-Haenzel estimates for sparse data were used for analyses in the usual frequency approach [25]. The unit of analyses was relapses. To test the robustness of the analyses we also restricted the analyses to the first relapse within each patient.

Spaces in the diary that were returned blank, both with regard to outcome and exposure measures, were handled as “missing” and excluded in the analysis. When calculating the usual frequency, we allowed information to be missing for one single day.

For statistical analysis, SAS version 9.1 (SAS Institute Inc. Cary, NC) was used.

### Ethical considerations

The study was approved by the Regional Ethics Committee in Stockholm, Karolinska Institutet, Dnr: 01-224, 04-813T. All data were handled anonymously.

## Results

The number of relapses during follow-up varied from 0 to 3, and a total of 42 relapses were identified. Twenty-five patients (50%) experienced one or more relapses, with the majority (52%) having one relapse. UC patients experienced more relapses than CD patients (Table 1).

**Table 1:** Baseline Characteristics of the Study Sample, Number of Patients (n) and Percentage (%)

	All patients (n = 60)	Relapse during follow-up (n = 25)	No relapse during follow-up (n = 35)
Male, n/%	24/40	10/36	15/43
Female, n/%	36/60	15/64	20/57
CD, n/%	29/48	9/36	20/57
UC, n/%	31/52	16/64	15/43
Age, mean (SD)	39 (1.31)	38 (12.53)	40 (15.91)
Localization of disease, n			
Proctitis	5	3	2
Proctosigmoiditis	8	5	3
Fulminant	18	8	10
Colon	21	6	15
Colon and small bowel	2	0	2
Small bowel	6	3	3
Duration of relapse, mean days (SD)		17 (25.74)	-

On scrutinizing the patients’ medical files, for the purpose of checking the identified relapses, we found that 18 of the relapses had not been recorded. Of the 19 recorded relapses, three were noted as continuous symptoms, one as an anal fissure, and in one case it was established that the patient did not have a relapse.

Of the 42 relapses, 19 (45.2%) were exposed to stress on the day before the onset of a relapse. Stress on one day increased the OR for relapse on the next day; 2.48 (95% CI 1.07 - 5.78) was found for the usual frequency analyses and 2.67 (95% CI 0.71 - 10.05) was found for the matched-pair interval analyses (Table 2). No increased effect estimates were found for hazard periods further than one day from onset (results not shown).

**Table 2:** Odds Ratio for Relapse in IBD After Exposure to Stress

Analytical approach	No. of subjects	No. of exposed cases during the day prior to onset	Odds ratio	95% confidence interval
Usual frequency	50	19	2.48	1.07 - 5.78
Matched-pair interval	50	19	2.67	0.71 - 10.05

Stratifying the analysis on level of stress showed an effect for high level of perceived stress (Table 3). When reporting “quite a lot” of stress, an OR of 4.8 (95% CI 1.09 - 21.10) was displayed. There was, however, no statistically increased risk of lower levels of perceived stress, although elevated effect estimates were also found for “some” stress. No one reported having “a lot” of stress during the day before the onset of a relapse. Stratification of the analyses with respect to diagnosis yielded an odds ratio of 7.33 (95% CI 0.94 - 57.33) for CD patients and 1.88 (95% CI 0.94 - 4.87) for UC patients.

**Table 3:** Odds Ratio (OR) for Relapse in IBD After Exposure to Stress During the Previous Day, 95% Confidence Interval (95% CI)

Analytical approach	Level of stress						
	1		2		3		4
	Odds ratio	95% confidence interval	Odds ratio	95% confidence interval	Odds ratio	95% confidence interval	
Usual frequency (95% CI)	1.38	(0.60 - 3.16)	2.57	(0.55 - 11.93)	4.8	(1.09 - 21.10)	-
Matched-pair interval	1.00	(0.32 - 3.10)	3.00	(0.31 - 28.84)	4.00	(0.45 - 35.79)	

If the analyses were restricted to the first relapse during follow-up, this did not alter the results.

## Discussion

The present study shows that perceived stress can act as a trigger for relapses in IBD. The major strength is the design. The patients were self-matched, thus acting as their own controls and were followed daily during six months. The case-crossover design eliminates all stable confounding factors. To our knowledge, no case-crossover study has been performed to examine the association between stress and relapses in IBD. However, the method has been used previously in order to examine the association between stress and other diseases, for example myocardial infarction [31], bacterial vaginosis [32], and Meniere’s disease [33].

Earlier studies have reported associations between stress and relapses in IBD [9, 10, 13-16, 18, 34]. However, they are inconclusive. In two review papers the authors highlight that the problem with earlier studies is due to the fact that there are differences between CD and UC patients regarding the effect of stress on relapses. A relationship has been found between stress and relapses in UC patients, but not in CD patients. On the other hand, it seems that depressive symptoms may cause a relapse in CD patients [18, 34, 35]. Nevertheless, these studies measured life events instead of perceived stress and did not follow the patients on a daily basis. In contrast, our study analysed CD and UC separately, and we are able to show that the trigger effect of stress seems to be higher for relapses in CD patients. However, our results should be considered with caution due to the small sample size. Our finding is in line with one study in which an increased risk of relapse associated with perceived stress was found in CD patients, but after removing the anxiety and depression components, the residuals of perceived stress were no longer associated with relapse [13]. Although, Bitton et al found that high stress influenced relapses in CD patients, and also reported an interaction between perceived stress and avoidance coping, which seemed to predict the time of relapse [14]. Bitton et al concluded that patients who score high on stress and adapt ineffective coping methods may benefit from counselling or stress management. However, intervention studies have been less successful in interrupting the link between stress and relapses [34]. Bernstein et al also report an association between perceived stress and relapse in IBD [9] by comparing patients that experienced a relapse with those who did not have a relapse. This study, however, differs in some important aspects from the present study as the patients were followed every third month and not on daily basis, and that the patients didn’t serve as their own control. Our results, thus, provide further evidence for the proposed trigger role of stress by addressing theses issues.

Another review paper found that the majority of the published studies have measured stress based on life events [10]. There were only a few controlled or prospective studies that reported a relationship between life events and relapses, whereas several other prospective studies found a relationship between stress and relapses using other measures of stress. There are different ways of measuring stress, for example stressful life events, stress diaries and specific questionnaires. Measuring subjective perception of stress may provide a better assessment of disease association. In the present study we used a diary to measure the patients’ perceived stress and levels of stress. An older study, including a small number of patients (6 with CD and 5 with UC), also used a diary to measure stress and found a relationship between daily stress and IBD symptoms in the following month [36].

Levenstein et al suggested that short-term stress does not trigger relapse in UC, but a long-term high level of perceived stress increases the risk over a period of months to years [15]. These results directly contradict the result in our study where we show a trigger effect of high levels of perceived stress during the day before a relapse. Both studies measure perceived stress but while the present study followed the participants daily for six months, Levenstein et al had a follow-up of 45 months and relapse status was monitored for up to 68 months.

One limitation in our study is the limited number of patients. Another limitation is the potential risk of recall bias. The participants were told to fill in the diary at the end of each day, but if they forgot they might have done it retrospectively. This retrospective reporting is, however, most likely if there was no variation in the reported variables. In spite of this potential for misclassification due to memory recall, one strength of the design is nevertheless that the participants were neither informed about the definition of relapse nor about the time periods of interest in the analyses. Furthermore, we have used a single-item measure of perceived stress. Although, there are several advantages of having a validated multi-items measure, our choice of a single item measure was made taking the considerations of the daily design of the study into account. It was important to use a measurement procedure that would be easy and quick for the participants, in order to not burden them with too extensive questionnaires. Earlier research has also showed that the single-item measure allows the person to take more personal characteristics into account when providing a response [30].

Relapses in our study were classified according to the patients’ symptoms. Eighteen out of 42 relapses had not been recorded in the patients’ medical files. However, this does not necessarily imply that these were not real relapses. Very often patients with IBD have relapses and do not contact their IBD clinic.

At the time of designing the present study the applications for electronic collection of day to day information was not well developed. In future studies we suggest either solely to rely on such a method or combining the two approaches. Electronic diaries offer many advantages over the pen and paper diaries such as ours used in the present study. Due to practical constraints we were not able to utilize this technology for the present study. Future studies of stress assessing self-reported data on daily basis should benefit from using electronic diaries.

Our study supports earlier findings regarding perceived stress as an important factor in triggering relapses in IBD. However, as this is the first time a case-crossover study has been conducted further investigations with larger power are needed to confirm our findings. This study illuminates the potential for preventing relapses among IBD patients by influencing stress levels and optimizes the clinical care of these patients.
